# Histological and ultrastructural evaluation of the early healing of the lateral collateral ligament epiligament tissue in a rat knee model

**DOI:** 10.1186/1471-2474-11-117

**Published:** 2010-06-13

**Authors:** Georgi P Georgiev, Nikolai K Vidinov, Plamen S Kinov

**Affiliations:** 1 Department of Anatomy, Histology and Embryology, Medical University Sofia, Bulgaria; 2 Department of Orthopedics and Traumatology, University Hospital Queen Giovanna, Sofia, Bulgaria

## Abstract

**Background:**

In this study, we evaluated the changes which occurred in the epiligament, an enveloping tissue of the ligament, during the ligament healing. We assessed the association of epiligament elements that could be involved in ligament healing.

**Methods:**

Thirty-two 8-month old male Wistar rats were used in this study. In twenty-four of them the lateral collateral ligament of the knee joint was surgically transected and was allowed to heal spontaneously. The evaluation of the epiligament healing included light microscopy and transmission electron microscopy.

**Results:**

At the eight, sixteenth and thirtieth day after injury, the animals were sacrificed and the ligaments were examined. Our results revealed that on the eight and sixteenth day post-injury the epiligament tissue is not completely regenerated. Till the thirtieth day after injury the epiligament is similar to normal, but not fully restored.

**Conclusion:**

Our study offered a more complete description of the epiligament healing process and defined its important role in ligament healing. Thus, we provided a base for new strategies in ligament treatment.

## Background

The incidence of knee ligament injuries has increased in recent years due to the general public's increase in sports activities [1-4]. Ligaments have been defined as dense bands of connective tissue that stabilize joints and guide joint motion [5,6]. After injury, ligaments do not heal by regeneration but by a formation of scar tissue similar to other wound healing models [6]. The normal and healing ligaments are composed of two major components: the extracellular matrix composed commonly of type I collagen and ligament cells [6,7]. Most studies investigated an insufficient repair process and tested different treatments regimes, including tissue engineering approaches, non-steroidal anti-inflammatory drugs, local corticosteroids, hyperbaric oxygen, growth factors, ultrasonic or electrical stimulation, laser therapy and also gene therapy [2,5,8,9]. However, mainly the animal models have gone into characterizing the extracellular matrix in both normal and injured ligaments [4,6,11-14], and only few of them have examined the ligament cells composed the enveloping tissue of the ligament, termed epiligament (EL) [6,8]. According to our opinion, the understanding of the healing process in the EL tissue could be essential in understanding the normal recovery in ligament and provide a basis for new treatment strategies. Therefore, we aimed to investigate both with light microscopy and transmission electron microscopy (TEM) the EL changes from the midsubstance of the lateral collateral ligament (LCL), which occurred through the early ligament healing and their possible role in restoration of the ligament.

## Methods

Thirty-two 8-month old male Wistar rats, with weight ranges of 350 - 400 g at the time of surgery, were used for this study after approval was obtained from the University Committee on Animal Resources. These rats were divided in four groups, each group including eight animals. The last group of animals underwent no transection and served as intact controls.

Twenty-four rats were anesthetized by intraperitoneal injection using a mixture of 5 mg/kg b.w. Xylasine (Bioveta, Czech Republic) and 45 mg/kg b.w. Calypsol (Ketamine, Gedeon Richter, Hungary). Their hind limbs were then shaven and washed with betadine solution. Under sterile conditions a small incision (10 mm) was made in their skin on the "femoro-fibular joint" (knee joint) of the left hind limb over the site of LCL. After skin incision, the overlying connective tissue was dissected to expose the knee's LCL. Then, a 1-mm gap in the mid-substance was surgically created and the gap was left without suturing. The transected ends were marked with 9-0 nylon monofilament suture. The skin incision was closed using 5-0 Ethibond suture. The right knee of other animals remained intact. The remaining eight rats were used as unoperated normal controls. After operation, the rats were allowed free cage activities. No infections or complications were observed in the twenty-four injury-induced animals. On the eight, sixteenth and thirtieth day after surgery, the animals were sacrificed with intracardiac injection of Thiopental (Sandoz GmbH, Austria). The unoperated controls were anesthetized, sacrificed as operated ones and then the same surgical approach was used. The injured ligaments were carefully removed without disturbing the scar region and were immediately fixed in 3% glutaraldehyde for 2 hours. The normal controls of the EL tissue were taken from the midsubstance of the LCL and were fixed as injured ligaments. Then both the controls and injured EL tissues were rinsed several times with 0.1% phosphate buffer to remove the fixative solution with subsequent incubation in a 1% osmium tetroxide for two hours was made. After that the pieces were dehydrated in EtOH (50, 70, 95, 100%). Next, the LCL scars were treated for 30 minutes with a 2:1 mixture of propylene oxide and epon. The pieces were embedded in Durcupan (Fluka, Buchs, Switzerland). Afterward all slices were processed with disectional microscope and cut with ultramicrotome (LKB, Stockholm-Bromma, Sweden). The scar regions were identified on semi-thin sections (for light microscopy) stained with 1% metilene blue, azure II and basic fuchsin. The EL tissue of the LCL was identified on semi-thin sections from the midsubstance in controls and gap region of the transacted ligament for operated animals (for light microscopy). The ultrathin sections (60 nm thick) were taken only from the EL gap region and from the midsubstance of the LCL epiligament tissue (for transmission electron microscopy) and both were contrasted with 2.5% uranyl acetate, lead nitrate, and sodium citrate.

## Results

**Figure 1:**
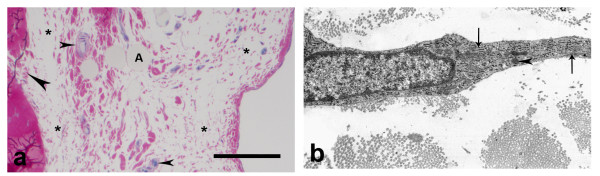
**Normal morphology of the external surface of the LCL epiligament tissue**. a) epiligament in controls with two types of layers (with star was marked the type, presented with fibroblasts, fibrocytes, surrounded by collagen fibers and rarely found blood vessels; the other type was between the marked parts of the previous composed of fibroblasts, fibrocytes, adiposities (A), mast cells, collagen fibers, nerves and vessels (small arrow head). The EL prolonged into endoligament (large arrow head) (light microscopy). Bar 100 μm; b) electron micrograph of fibroblast in the intercellular matrix with large nucleus, collagen fibers, rough endoplasmic reticulum (arrows) and single lysosome (arrow head) × 9000.

Normal rats' EL structure has been previously described by us [15]. Histologically there were two types of layers in the EL (Fig. 1a). The first type is composed of packed fat cells, mast cells, fibroblasts, fibrocytes, collagen fibers, nerves and vessels. The second layer is composed only of fibroblasts, fibrocytes, surrounded by collagen fibers and rarely found blood vessels. The EL is relatively abundant of blood vessels and is relatively better vascular than the ligament substance. TEM revealed in the EL different types of fibroblasts: with spindle-shape, spinous-shape, elongated and irregular form. They had large nucleus, well developed granular endoplasmic reticulum, poorly developed Golgi apparatus, single spherical mitochondria and single lysosomes (Fig. 1b). Collagen fibers in the midsubstance of the external surface of the LCL EL had uniformly small diameters and were organized in bundles with different orientations, in contrast to the parallel collagen fibers in the ligament. There were also chaotically orientated small groups of collagen fibers. In the EL both myelinated and unmyelinated nerve fibers were detected.

**Figure 2:**
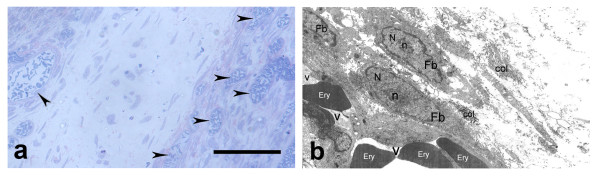
**Epiligament scar at the eight day after injury**. a) epiligament scar tissue composed of plump fibroblasts (colored in blue), disorganized matrix with collagen fibers (colored in red) and intensive angiogenesis (arrow heads) (light microscopy). Bar 100 μm; b) electron micrograph of fibroblasts (Fb) in the intercellular matrix with large nucleus (N) and enormous nucleolus (n), collagen fibers (col), blood vessels (v) with erythrocytes (Ery) on the eight day after injury × 3500.

**Figure 3:**
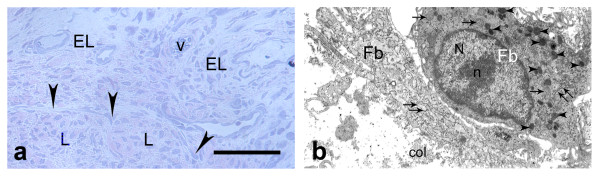
**Epiligament-ligament scar at the eight after injury**. a) deep part of the epiligament (EL), consisting fibroblasts (colored in blue), collagen fibers (colored in red) and vessels (v), penetrating into endoligament tissue (arrow heads), which envelop collagen bundles in the ligament scar (L) (light microscopy). Bar 100 μm; b) electron micrograph of fibroblasts (Fb) and its organelles - nucleus (N), nucleolus (n), mitochondria (arrows), lysosomes (arrow heads), rough endoplasmic reticulum (double arrows); and collagen fibers (col) in the intercellular matrix on the eight day after injury × 7000.

The histological results demonstrated at the eight day after injury (Fig. 2a; Fig. 3a), a substantial bridge of clearly distinguishable granulation of the EL tissue connecting the transected edges. The transected regions of the EL were characterized with hyper-cellularity, presented mainly from fibroblasts and progenitor cells. These cells had an oval basophilic nucleus and their cytoplasm appeared vitreous and was only lightly stained. The numerous cells localized in the deep part of the EL substance prolonged to the endoligament enveloping the collagen fibres of the ligament. Intensive angiogenesis was observed in the EL scar region. It was a result of a vascular response in the chaotic arborization of capillaries from larger blood vessels within the healing region. As a whole the described two types of layers could not be distinguished.

TEM analysis at the eight day post-injury (Fig. 2b; Fig. 3b) further showed different types of fibroblasts - elongated, spinous-shape and also irregular in forms. They had abundant and irregularly branched cytoplasm with short plasma membrane processes. Their large nucleuses, picnotic in some of the cells displayed a very delicate chromatin structure, clearly visible heterochromatin dominated in the peripheral nuclear area, and an enormous prominent nucleolus. The cytoplasm contained considerable amount of free ribosomes, polysomes, expanded rough endoplasmic reticulum and poorly developed Golgi apparatus. There were also large fibroblasts with well-developed rough endoplasmic reticulum, which filled most of intracellular space. Spherical mitochondria with clearly visible cristae, phagocytic vacuoles and an abundance of lysosomes presented with uniformly granular, electron-dense appearances were observed. Intensive angiogenesis were presented with numerous blood vessels in the EL substance. In the intercellular space, chaotically rare arranged collagen fibres were detected. There was also small amount of collagen fibers, organized in bundles with different orientations. However, some of them included fibers with altered characteristics and their normal striation pattern of lighter and darker bands were disturbed.

**Figure 4:**
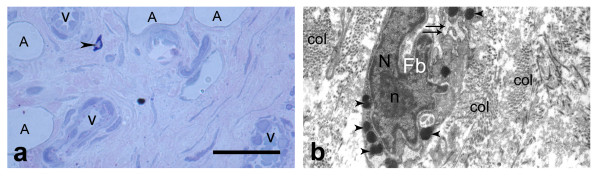
**Epiligament scar at the sixteenth day after injury**. a) epiligament (EL), consisting fibroblasts (colored in blue), collagen fibers (colored in red), mast cell (arrow head), adiposities (A) and vessels (v) (light microscopy). Bar 20 μm; b: electron micrograph of fibroblast (Fb) with large nucleus (N), nucleolus (n), lysosomes (arrow heads) and rough endoplasmic reticulum (double arrows); and collagen fibers (col) in the intercellular matrix on the sixteenth day after injury × 9000.

**Figure 5:**
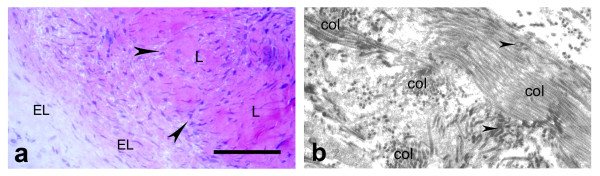
**Epiligament-ligament scar at the sixteenth day after injury**. a) epiligament (EL), consisting fibroblasts (colored in blue), collagen fibers (colored in red) prolonging to endoligament (arrow heads) enveloping the collagen bundles of the ligament (L) (light microscopy). Bar 50 μm; b) electron micrograph of collagen fibers in the EL organized in bundles with different orientations (col) and collagen fibers between bundles, some of them with irregular striation pattern (arrow heads) and also included in the bundles of regularly orientated collagen fibers on the sixteenth day after injury × 12000.

On the sixteenth day after injury (Fig. 4a; Fig. 5a) the granulation margins in the EL were less distinguishable due to intermingling of new collagen fibers with uninjured fibers. The EL tissue was also hyper-cellular, but less than in previous period. The fibroblasts and progenitor cells in the deep part of the EL also migrated in the endoligament, but they were better organized and compacted. In the regenerative zone of the EL clusters of adipocytes with irregular forms and variable sizes were observed. The angiogenesis in the EL tissue began to diminish. As in previous period the two types of layer in the EL could not be discovered.

Ultrastructurally, at the sixteenth day post-injury (Fig. 4b; Fig. 5b) fibroblasts in the scar region had large, lobulated, picnotic in some cells nuclei, but their nucleoli were smaller in diameter and decreased in number, than in previous period. The organelles, such as the granular endoplasmic reticulum and Golgi apparatus did not changed their characteristics. However, the lysosomes and spherical mitochondria decreased in numbers and size. Phagocytic vacuoles in the cytoplasm were detected. There were also fibroblasts with normal characteristics as in controls. The adipocytes had a large vacuole and eccentric, flatten nuclei surrounded by a basal lamina. The number of blood vessels in the EL tissue decreased. Their intima consisted of rough layers of endothelial cells lining the vessels interior surface and had fine processes orientated to the lumen of the vessel. These cells rested on a well-distinct basal lamina. The number of collagen fibers increased in this period. There was collagen fibers with transverse striation organized in bundles with different orientations. However, there were collagens fibers with altered characteristics between the separate bundles as well as included within them. Chaotically orientated small groups of collagen fibers between bundles were also detected.

**Figure 6:**
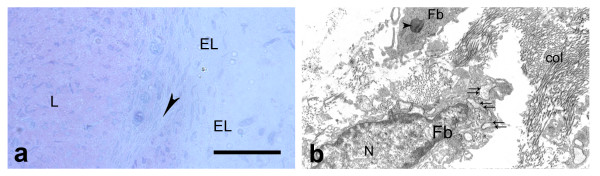
**Epiligament-ligament scar at the thirtieth day after injury**. a) epiligament (EL) scar tissue, consisting fibroblasts (colored blue), collagen fibers (colored red) and ligament scar (L) (light microscopy). Bar 50 μm; b) electron micrograph of fibroblasts (Fb) and its organelles - nucleus (N), lysosome (arrow head), rough endoplasmic reticulum (double arrows); and collagen fibers (col) in the intercellular matrix on the thirtieth day after injury × 7000.

**Figure 7:**
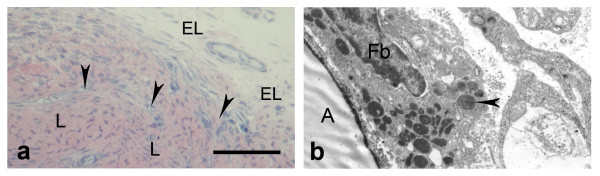
**Epiligament-ligament scar at the thirtieth day after injury**. a) epiligament (EL) scar tissue prolonging in endoligament (arrow heads) enveloping the collagen bundles of the ligament scar (L) (light microscopy). Bar 50 μm; b) electron micrograph of fibroblast in the intercellular matrix with numerous lysosomes (arrow head) and adipocyte (A) × 7500.

On the thirtieth day after injury (Fig. 6a; Fig. 7a) the healing process advanced and cells of the EL infiltrated most of the LCL scar, while collagen disorganization subsided. The EL tissue was similar to controls and was composed of fibroblasts, fibrocytes, adipocytes, mast cells and reduced number of vascular network, than in previous period.

TEM at this period (Fig. 6b) revealed different types of fibroblasts with large nucleuses: elongated fibroblasts, spindle-shaped, spinous-shaped and fibroblasts with irregular form. Some of them had well-formed short cytoplasmic processes. In the nucleus of the fibroblasts there was fine granular chromatin, denser near the inner nuclear membrane structures and one nucleolus. The electron-dense, finely granulated cytoplasm consisted of free ribosomes, well-developed granular endoplasmic reticulum, mitochondria, phagocytic vacuoles, poorly developed Golgi apparatus and one or rarely two lysosomes were observed. Extremely rare fibroblasts with numerous lysosomes were also found (Fig. 7b). Collagen fibers in the intercellular space increased in number. They have uniformly small diameters and were organized in bundles with different orientations. Occasionally, fibers with abnormal striation pattern were discovered. There were also small groups of collagen fibers chaotically orientated.

## Discussion and Conclusion

Ligaments' healing involves a complex, coordinated series of events that form a neo-ligament which is more scar-like in character than the native tissue [8]. Numerous studies have investigated the healing process of the collateral ligaments of the knee in animal models [1,2,5,6,10,16,17]. However, very little is known about changes which occurred in the EL after ligament injury [6,8]. We presented the results from the first detailed ultrastructural study of the early events in EL healing. We presented the modification of the EL scar throughout the initial 30 days after injury of the ligament and described different types of fibroblasts within the EL healing.

The EL structure is quitе different from the ligament substance [6,15,18]. The ligaments are described as poorly vascularized connective tissue, composed of fascicles [6]. These fascicles are formed by longitudinal groups of collagen fibers [7]. Each fascicle appears hypocellular and the cells are aligned interspersed between bundles of collagenous fibers [6,7]. In contrast the EL is more cellular than the ligament and is composed of different types of cells, and contains abundant blood vessels and nerves [6,15,18]. Interestingly, when cells are grown in vitro, they do not orientate themselves parallel to the long axis of the tension, but away from it [6]. This orientation is strikingly similar to that found in the EL in vivo, where the cells are orientated perpendicular to the longitudinal axis of the ligament [6]. This suggests that the EL might also be tensile bearing [6].

Due to the characteristics of the EL tissue Lo et al. [6] supposed that the EL may be the major source of cells that made up the ligament scars during ligament healing. According to Chowdhury et al. [19] the EL cells closely resemble the fibroblastic cells which compose ligament scar tissue. Chamberlain et al. [8] also stated that ligament injury stimulates in the EL the release of variety of cell types, including neutrophils and mitotic cells till the 5 days post-injury. Circulating macrophages, resident macrophages, T lymphocytes, hematopoietic cells, vascular endothelial growth factor with crest between 5 to 9 days post injury were also established in the EL. These cells and blood vessels in the EL once localized in the ligament body proliferate and migrate. According to Chamberlain et al. [8] the process of creeping substitution by the developing granulation tissue results in cells localizing from the healing region and into the healing edges. The presence of EL cells within the ligament had a number of other implications [6]. As with the epitenon, the EL cells may be involved in differentiation, phagocytosis and collagen synthesis, and thus take part in ligament healing [6].

Our light microscopic study on the eight day after injury revealed that the scar regions were characterized with hyper-cellularity and intensive angiogenesis. Numerous cells in the deep part of the EL substance migrated in the endoligament enveloping the collagen fibres of the ligament. This was in contrast to relatively small number of cells in the EL presented near the ligament substance in unoperated animals. TEM observations revealed active fibroblasts with short plasma membrane processes. Their large nucleuses had an enormous prominent nucleolus, typical characteristic of cells that were actively synthesizing proteins. The cytoplasm contained considerable amount of free ribosomes, polysomes, expanded rough endoplasmic reticulum also a sigh for active protein synthesis. High incidence of lysosomes in fibroblasts of injured animals, in contrast to controls exhibited their higher phagocytic activity. The detected high amounts of spherical mitochondria opposed to uninjured animals were a characteristic of a more intense metabolic activity. Intensive angiogenesis was presented with increasing number of blood vessels in the EL substance indicating late inflammation and early proliferative phase. Chaotically arranged single or rarely small groups of collagen fibres did not reveal a well-presented restoration of the EL. On the sixteenth day after injury the light microscopy research presented similar characteristics as in previous period, but the granulation margins in the EL were less distinct and the scar region appeared to be more organized. Deep part of the EL was also hypercellular, differently to controls and these cells also migrated in the endoligament enveloping the collagen fibres of the ligament. The fibroblasts in the scar region also had large nuclei and abundant rough endoplasmic reticulum as in previous period. The number of lysosomes and mitochondria decreased, but was higher than controls. All these characteristics indicated less phagocytic activity and less activation of fibroblasts. In the regenerative zone of the EL there was single or clusters of adipocytes representing a new packing material for EL tissue. However, they had irregular form and varied in size compared to unoperated animals consisted single adipose cells with spherical or polyhedral form when they are closely packed. The number of blood vessels in the EL tissue decreased presenting the remodeling phase. The collagen fibers in this period were also organized in bundles with different orientations and damaged collagen fibers, between and included in them, but more regular than in the previous period. On the thirtieth day after injury the healing process advanced and cells of the EL infiltrate most of the LCL scar, while collagen disorganization subsided. The EL tissue was similar to controls and was composed of fibroblasts, adipocytes, mast cells and reduced number of vascular network, but not fully restored. The cells in the deep part of the EL decreased in numbers as in controls. TEM presented mostly single lysosomes in the EL's fibroblasts, similar to controls. However, incidentally fibroblast with numerous lysosomes and single fibers with damaged characteristics were discovered, presented not completely restoration of the EL tissue.

Light microscopic investigations revealed that the general cellular morphology of EL was similar to that seen in synovium [19]. This is consistent with the hypothesis that the EL is a specialized form of synovium [6].

Limitations of the current animal model existed and should be noted. First, all injuries were induced by scalpel transaction, a method that is not an ideal simulation of common clinical injuries [8,11]. Second, the dehydration processes during sample preparation of ligaments can induce a shrinkage artifact. Because this study was not quantitative, shrinkage errors were less important and probably had little effect on our observations. Third, we studied only the midsubstance of the EL of the LCL. There were studies, which revealed that injury location affects ligament healing [10].

In conclusion, this study illustrates for the first time the ultrastructural changes of the early reparation of the EL tissue during first month of ligament healing. As described, the EL is the main source of fibroblasts, progenitor cells and blood vessels that proliferated and infiltrated within the ligament body via the endoligament during ligament healing. Therefore, detailed knowledge of the EL as well as its normal morphology and its restoration during ligament healing is essential to get a better understanding of the normal healing process and thus propose optimal treatment regimes.

## Competing interests

The authors declare that they have no competing interests.

## Authors' contributions

GPG and NKV conceived the study. GPG wrote the manuscript, design the study, prepared the light and transmission microscopy and analyzed the results. NKV and PSK helped in analyzing the result section and preparing the manuscript. All authors have read and approved the final manuscript.

## Pre-publication history

The pre-publication history for this paper can be accessed here:

http://www.biomedcentral.com/1471-2474/11/117/prepub
